# Oxidation Protective Hybrid Coating for Thermoelectric Materials

**DOI:** 10.3390/ma12040573

**Published:** 2019-02-14

**Authors:** Francesco Gucci, Fabiana D’Isanto, Ruizhi Zhang, Michael J. Reece, Federico Smeacetto, Milena Salvo

**Affiliations:** 1 School of Engineering and Material Science, Queen Mary University of London, London E1 4NS, UK; ruizhi.zhang@qmul.ac.uk (R.Z.); m.j.reece@qmul.ac.uk (M.J.R.); 2 Nanoforce Technology Limited, London E1 4NS, UK; 3 Department of Applied Science and Technology, Politecnico di Torino, 10129 Turin, Italy; fabiana.disanto@polito.it (F.D.); milena.salvo@polito.it (M.S.); 4 Department of Energy, Politecnico di Torino, 10129 Turin, Italy; federico.smeacetto@polito.it

**Keywords:** Thermoelectrics, oxidation resistance, hybrid-coating

## Abstract

Two commercial hybrid coatings, cured at temperatures lower than 300 °C, were successfully used to protect magnesium silicide stannide and zinc-doped tetrahedrite thermoelectrics. The oxidation rate of magnesium silicide at 500 °C in air was substantially reduced after 120 h with the application of the solvent-based coating and a slight increase in power factor was observed. The water-based coating was effective in preventing an increase in electrical resistivity for a coated tethtraedrite, preserving its power factor after 48 h at 350 °C.

## 1. Introduction

Thermoelectric materials are able to convert thermal gradient into electricity and recover energy from waste heat [1,2]. They are, usually, semiconductors or intermetallics, often containing elements such as Mg, Pb, Te, Bi, Mn, Ge, Si, Sb, Co or In [3,4,5,6,7]. 

A considerable effort has been made to produce materials with high figure of merit, and several compounds have been identified and improved using different approaches: doping elements; composites; nanostructuring; and mesostructuring [3,4,8]. The effect of conventional synthesis and sintering techniques have been evaluated [9] and innovative methods are constantly under development [10,11,12,13,14]. One of the main challenges in the thermoelectrics field is the identification of efficient materials that are inexpensive, easy to be produced, and formed of earth-abundant and environmentally friendly elements. In this respect, magnesium silicide [15] and tetrahedrite [16] are considered attractive and sustainable candidates for n and p-type thermoelectrics, respectively. One important aspect for the development of high temperature thermoelectric generators is their long-term stability in air at high temperature [17]. 

Magnesium silicide is a semiconductor of the Mg_2_X (X = Si, Ge, Sn and Pb) compounds family. It possess an anti-fluorite structure with a bandgap of 0.784 eV [18]. It can be doped to achieve good thermoelectric properties (ZT of 0.86 at 862 K with Bi-doping [19]) but it is limited by its relatively high thermal conductivity. N-type solid solutions of Mg_2_Si with Mg_2_Sn [20,21,22,23] and/or Mg_2_Ge [24,25] have been produced in an attempt to reduce their thermal conductivity. One of the best values has been reported for Mg_2.08_Si_0.364_Sn_0.6_Sb_0.036_ (ZT of 1.5 at 723 K [26]).

Magnesium silicide and its solid solutions are prone to oxidation above ~400 °C; Skomedal et al. [27] reported breakaway oxidation behavior for Mg_2_Si_1−x_Sn_x_ (0.1 < x < 0.6) at temperatures above 430 °C. Sondergard et al. [28] showed the substantial stability in air of Mg_2_Si_0.4_Sn_0.6_ and Mg_2_Si_0.6_Sn_0.4_ up to 400 °C when the material has a high relative density. Yin et al. [29] reported the oxidation behavior of Sb-doped Mg_2_Si_0.3_Sn_0.7_ (360–720 h) to prevent the decomposition they proposed and studied the effect of BN spray-coating which was effective up to 500 °C. Tani et al. [30] studied the effect of magnetron sputtered β-FeSi on Mg_2_Si, observing that it improved the oxidation resistance up to 600 °C.

Tetrahedrite (Cu_12_Sb_4_S_13_) is a ternary I-V-VI semiconductor, which has a complex crystal structure with a large number of atoms per unit cell, helpful in providing low thermal conductivity, and a high band degeneracy (1.7 eV) [31,32] due to its highly symmetric crystal structure, which is useful for improving the power factor [33]. It has a sphalerite-like structure with 58 atoms arranged in a high symmetry cubic cell (I43m) made of CuS_4_ tetrahedra, CuS_3_ triangles and SbS_3_ pyramids [34]. This structure, with lone-pair electrons on Sb sites is the origin of the low lattice thermal conductivity [32], which is shared by other compounds in the Cu-Sb-S system [35] such as chalcostibite [36] (CuSbS_2_), famatinite [37,38] (Cu_3_SbS_4_) and skinnerite (Cu_3_SbS_3_) [39]. Naturally, tetrahedrite occurs with the composition Cu_12−x_M_x_(Sb,As)_4_S_13_, which is a solid solution of As rich tennantite (Cu_12_As_4_S_13_) and Sb rich tetrahedrite (Cu_12_Sb_4_S_13_). The literature related to this material shows the best Thermoelectric properties are achieved by replacing Cu^2+^ atoms with Zn: ZT is 0.6 (at 400 ° C) for an un-doped sample, and increases up to 0.9 (at 447 °C) with Zn substitution due to a reduction of the thermal conductivity [40]. 

Tetrahedrite has limited thermal stability and is subjected to sulphur loss. Braga et al. [41] reported the phase decomposition of Cu_12_SbS_13_ at around 795 K. Barbier et al. [42] confirmed the same phase transformation at 803 K corresponding to a weight loss due to Sulphur volatilization. Nevertheless, Chetty et al. [16] reported that tetrahedrite is usually stable only up to 600 K, and that the overall stability may increase or decrease depending on the dopants. The oxidation behavior of a tetrahedrite was tested only by Gonçalves et al. [43]. They discovered the formation of a Cu_2−x_S surface barrier, which decreases the corrosion rate at 275 °C acting as a weak passivation layer. Nevertheless, this layer was not effective at 350 °C and 375 °C because of the simultaneous action of sulphur sublimation.

Second phases were present in all of the samples at the end of the test, evidencing the need of an effective protective coating, but no studies have been carried out to identify a suitable one for this thermoelectric material. In general, there is a relatively small body of literature that is concerned with oxidation protective coatings for TE modules.

The oxidation of the surface of the thermoelectric degrades the power generation and significantly limits the long-term reliability and efficiency of TE modules. For this reason the application of an oxidation resistant coating is needed to improve thermoelectric properties.

In this work, we investigated the potential of two commercial hybrid (ceramic-polymer) coatings with nominal temperature resistance up to 590 °C. To evaluate their effectiveness, we tested the properties of Mg_2_Si_0.487_Sn_0.55_Sb_0.13_ and Cu_11.5_Zn_0.5_Sb_4_S_13_ as sintered and after aging in air, with and without the coatings. The low curing temperature (250 °C) of these resins is a great advantage; in fact, glass-ceramics coatings would require deposition temperatures too high for tetrahedrites (for example, a Higher Manganese Silicides coating was prepared at 700 °C [44]). Moreover, the coating procedure does not require the need of expensive equipment (such as magnetron sputtering) making it more appealing for actual device production.

## 2. Materials and Methods 

Mg_2.1_Si_0.487_Sn_0.5_Sb_0.13_ (Mg-silicide) powders were provided by European Thermodynamics Ltd (Leicester, UK). Powders were then sintered into 30 mm diameter discs using a Spark Plasma Sintering furnace (FCT HPD 25; FCT Systeme GmbH, Rauenstein, Germany). The sintering of Mg-silicide was carried out at a temperature of 750 °C with a heating and cooling rate of 100 °C/min, a dwell time of 5 min and a pressure of 50 MPa. 

Cu_11.5_Zn_0.5_Sb_4_S_13_ (THD) was prepared starting from single elements powders: Cu (Alpha Aesar, 150 mesh, purity 99.5%), Sb (Alpha Aesar, 100 mesh, purity 99.5%), S (Sigma Aldrich, 100 mesh, purity reagent grade) and Zn (Sigma Aldrich, ≥ 99%). They were weighted in the appropriate stoichiometry and sealed in a stainless steel jar in an argon filled glove box, processed in a ball milling machine (QM-3SP2, Nanjiing University, China) employing stainless steel balls at 360 rpm for 96 h, with a ball to powder ratio of 30:1. The sintering of tetrahedrite was carried out at a temperature of 400 °C with a heating and cooling rate of 50 °C/min, a dwell time of 5 min and a pressure of 50 MPa. The density of the pellets was measured using the Archimede’s method. Each pellet was cut into bars having square base of 3 mm per side and 10 mm height. 

After preliminary tests, a solvent-based resin (CP4040-S1) was chosen for Mg-Silicide and a water-based resin (CP4040) for tetrahedrite, both purchased from AREMCO SCIENTIFIC COMPANY (Los Angeles, USA). They were applied using a foam brush and subsequently cured in a tubular furnace (Carbolite Gero STF/180, Neuhausen, Germany) for 45 min at 250 °C with a heating and cooling rate of 1.6 °C/min. Aging tests was performed in a muffle oven (Manfredi OVMAT 2009, Pinerolo, Italy) in air at a temperature of 500 °C for 120 h (for Mg-silicide) and at 350°C for 48h (for THD) with a heating rate of 1.2 °C/min. The choice of oxidation temperatures was guided by previous literature and the potential operating temperatures of the materials. Both Mg-Silicide and tetrahedrite are oxidized in air at these temperatures without being subjected to any phase transformations and their properties are near their optimum values. The tests would provide an initial benchmark for the tested hybrid coatings. 

XRD data were collected using X’Pert Pro MRD diffractometer with Cu Kα radiation (PANalytical X’Pert Pro, Philips, Almelo, The Netherlands, with the aid of the X-Pert HighScore software) and the different phases were identified using the JCPDS data base. Field emission scanning electron microscope (FE-SEM, Merlin electron microscope, ZEISS, Oberkochen, Germany) and energy dispersive X-ray Spectroscopy (EDS, ZEISS Supra TM 40, Oberkochen, Germany)were used to characterize the microstructure morphology and chemical composition of uncoated and coated samples, before and after ageing. The measurements of the electrical properties were carried out using a Linseis LSR-3 (Linseis Messgeraete GmbH, Selb, Germany) with Pt thermocouples and electrodes. The oxide layer of the aged samples was removed before measuring their properties.

## 3. Results and Discussion

### 3.1. Mg-Silicide (Solvent-Based Coating)

**Figure 1:**
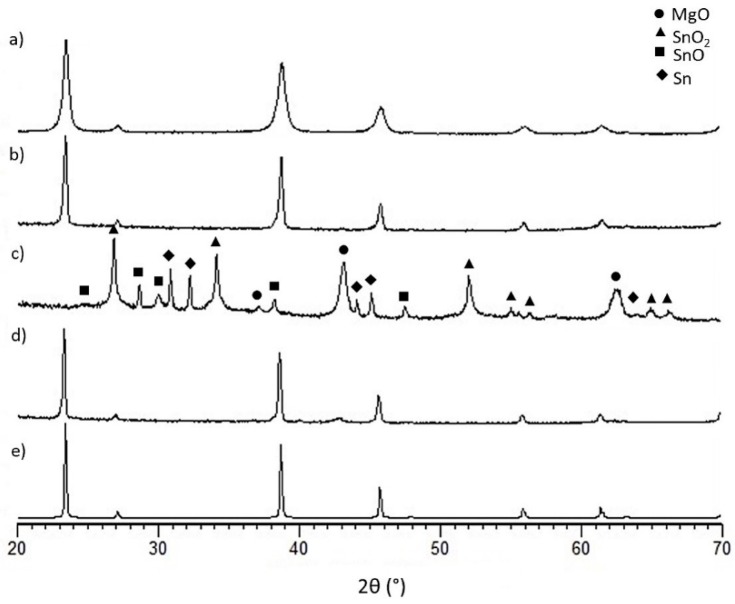
XRD spectra for (**a**) Mg-silicide powders (**b**) as sintered sample (**c**) aged Mg-silicide at 500 °C for 120 h without coating (**d**) aged Mg-silicide at 500 °C for 120 h with coating and (**e**) PDF card of Mg_2_Si_0.4_Sn_0.6_ (n. 01-089-4254).

Ball milling of the elemental powders effectively produced a single phase solid solution (Mg_2_Si_0.4_Sn_0.6_) of Mg_2_Si and Mg_2_Sn, and no peak splitting was observed in the XRD pattern (Figure 1a). After sintering, no phase separation was visible in the XRD pattern and the peaks simply became sharper (Figure 1b). 

The density of the as sintered sample was about 96% of the theoretical one.

**Figure 2:**
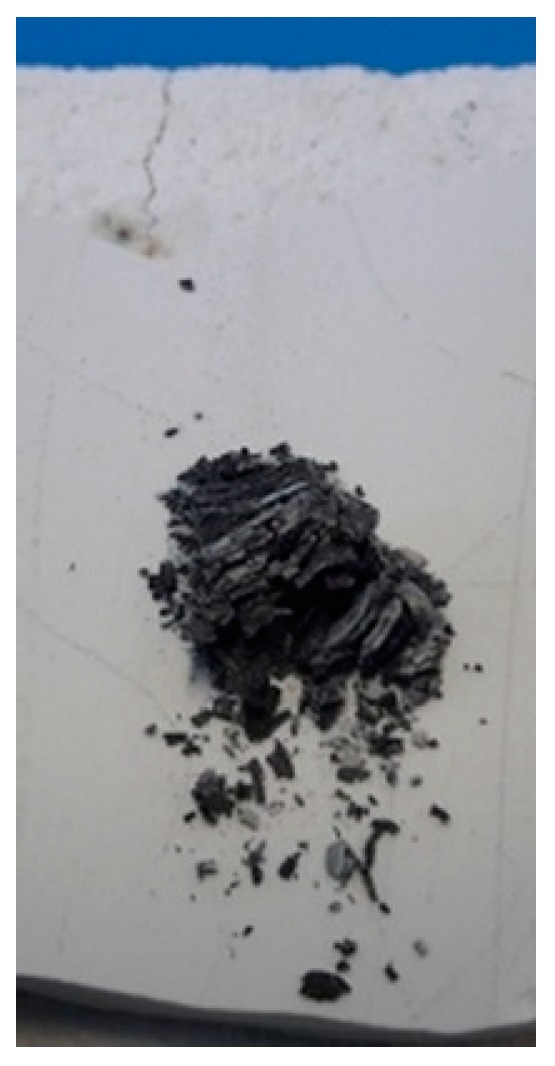
Mg-silicide uncoated after aging at 500 °C for 120 h, in air.

The aging at 500 °C for 120 h in air had a very clear effect on the uncoated sample; it was completely burned and turned into powder (Figure 2). The coated sample did not suffer such a catastrophic effect despite the fact that the applied coating was damaged at the edges.

The XRD pattern of the uncoated sample after aging (Figure 1c) shows the decomposition of Mg_2_Si_0.4_Sn_0.6_ into a mixture of compounds (MgO, SiO, SnO_2_ and Sn), as already observed by Skomedal et al. [27].

**Figure 3:**
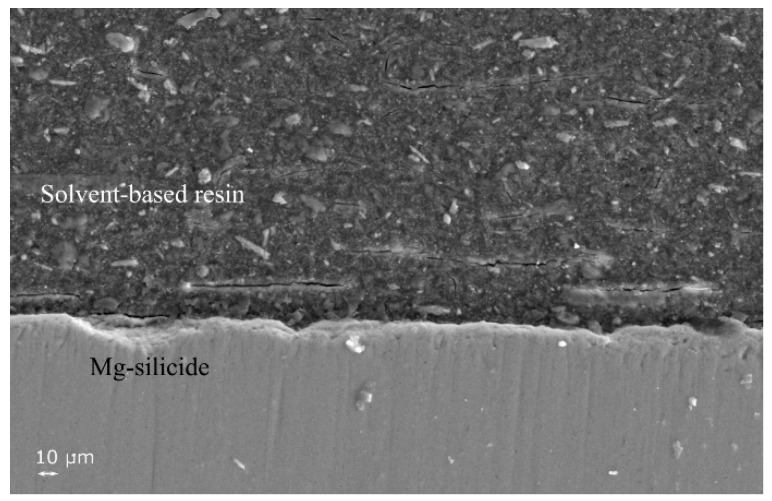
Cross sectional SEM image of coated sample after curing at 250 °C for 45 min under flowing Ar.

Figure 3 shows the cross-section of a coated sample after the curing. The interface between Mg-silicide and the hybrid coating is continuous, without cracks. However, the coating shows a few cracks parallel to the surface; this was likely due to a mismatch in CTE between the matrix and ceramic filler or more likely an effect due to solvent evaporation during curing, with consequent shrinkage effects. The thickness of the layer was found to be about 30–100 µm, being thinner at the edges.

**Figure 4:**
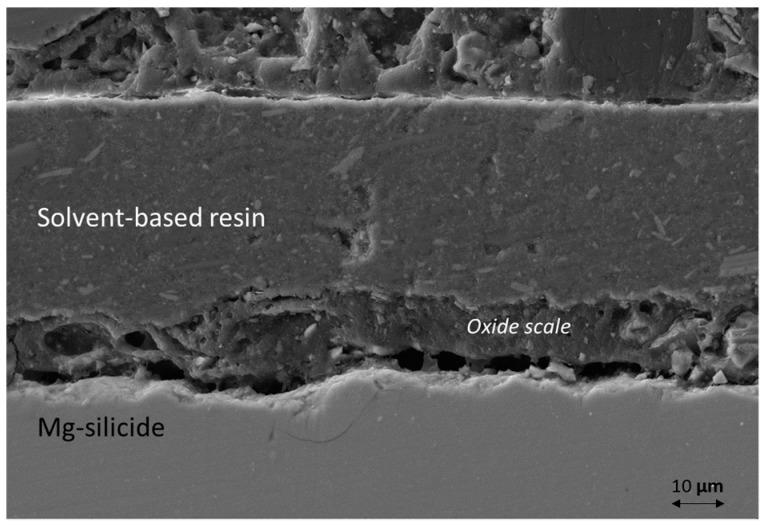
Cross sectional SEM image of solvent-based resin coated Mg-silicide after aging at 500 °C for 120 h, in air.

After aging for 120 h at 500 °C in air, the coated sample did not experience significant oxidation, and was still mainly composed of a single phase (Figure 1d). However, a small amount of MgO was present and the coating showed cracks on the edges and peeled off in some areas. Figure 4 shows the cross-section of a coated Mg-silicide after the oxidation test: an uneven oxide scale formed on the TE surface, thus indicating that the coating effectively reduced the oxidation reaction rate compared to the uncoated sample but did not prevent it as an oxide layer growing at the coating/sample interface.

**Figure 5:**
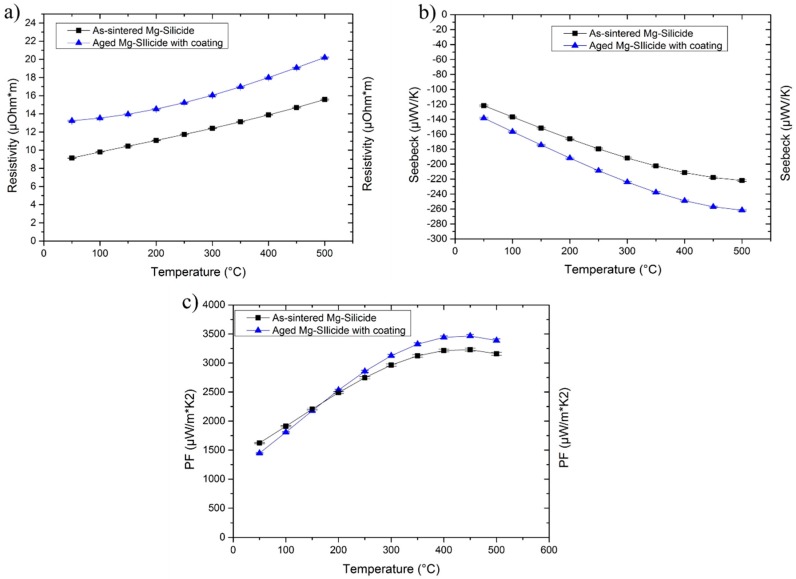
Temperature dependence of the: (**a**) Electrical resistivity; (**b**) Seebeck coefficient; and (**c**) Power Factor for Mg-silicide as sintered and coated after ageing at 500 °C for 120 h, uncoated could not be measured. (For all electrical measurements, the outer layers were removed coated sample to reveal pristine silicide onto which electrical contacts have been made).

The comparison between the electrical properties of the as-sintered sample and coated sample after ageing is a useful tool to understand the effectiveness of the coating (Figure 5). 

It was clearly impossible to measure the properties of the uncoated sample after the oxidation test since it was completely destroyed (Figure 2). The initial properties of the sample are comparable to those of a similar composition reported in the literature [20,22]. The electrical resistivity (ρ) of the coated sample after aging was increased by about 50%, while the Seebeck coefficient (S) increased by about 10%. Different stoichiometric ratio of Mg_2_Si and Mg_2_Sn as well as Mg vacancies or interstitial (due to the fact that Mg diffuses towards the surface) can influence the electronic properties [15,22,45,46].

The coating provides a very good degree of protection of Mg-silicide, against a complete burning of the Mg-silicide substrate.

From the data, the power factor (S^2^/ρ) of the coated samples seems to be slightly increased. On the other hand, it is also clear that the effectiveness is time-limited and longer ageing times may likely determine the total oxidation of the sample, as occurred for the uncoated sample.

Further work is needed to fully understand the implications of defects or non-homogeneous areas through the coating.

Further work should focus on the production of a homogeneous coating with a controlled optimal thickness, which should prevent the surface damage and remove easy path for oxygen diffusion. Due to the sample shape, it was not possible to evaluate thermal conductivity and, therefore, ZT. 

### 3.2. Tetrahedrite (Water-Based Coating)

**Figure 6:**
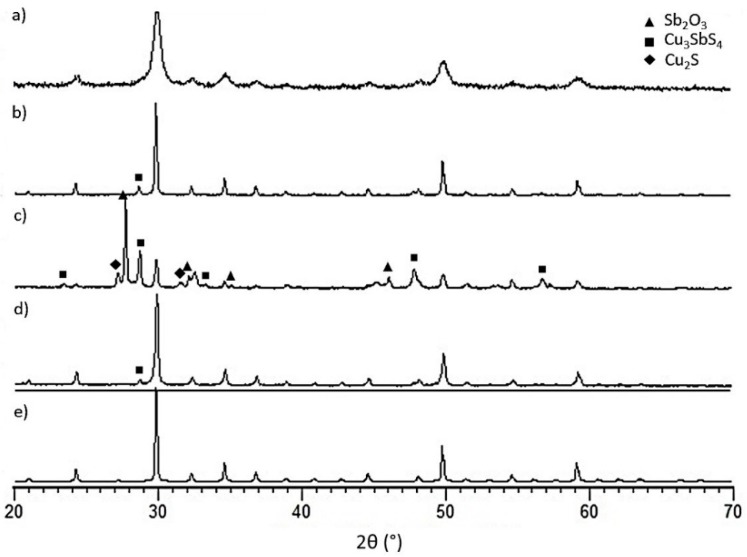
XRD spectra for (**a**) Tetrahdrite powders (**b**) as sintered sample (**c**) aged THD at 350 °C for 48 h without coating (**d**) aged THD at 350 °C for 48 h with coating and (**e**) Cu_12_Sb_4_S_13_ PDF Card n.00-024-1318.

The Ball milled powders produced starting from single elements consisted of single phase Cu_12_Sb_4_S_13_ (PDF Card n.00-024-1318) (Figure 6a). The density of the as sintered THD measured with the Archimede’s method was found to be about 98% of the theoretical density. The XRD pattern of the as-sintered THD (Figure 6b) confirmed that the main phase is Cu_12_Sb_4_S_13_ with a minor amount of Cu_3_SbS_4_ (Famatinite PDF Card n. 01-071-0555). 

**Figure 7:**
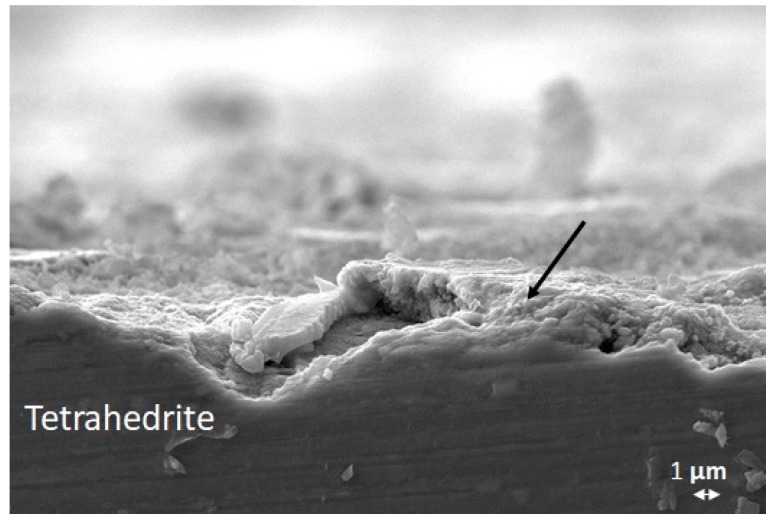
SEM image of cross-section of uncoated THD after ageing at 350 °C, dwelling time 48 h, in air.

After the ageing at 350 °C for 48 h in air, the uncoated THD was oxidised with a darker surface than the as-sintered sample. Cross sectional SEM images of the uncoated THD (Figure 7) shows the formation of an inhomogeneous layer (around 3–5 µm) on the whole surface of the thermoelectric. The point indicated with the black arrow can be attributed to antimony oxide.

**Figure 8:**
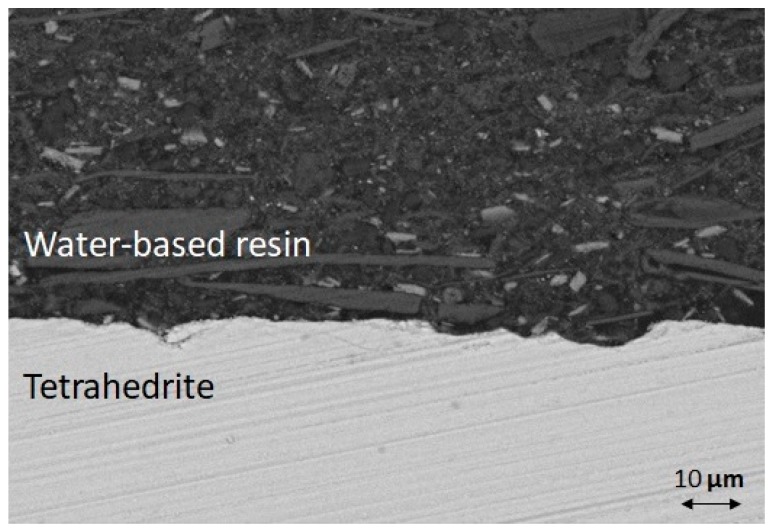
SEM image of cross-section of water-based resin THD coated after curing at 250 °C for 45 min under flowing Ar.

The XRD analysis of the uncoated sample surface after ageing (Figure 6c) shows that the main phase was Sb_2_O_3_ (PDF Card n. 00-043-1071), confirming the SEM analysis, with the presence of Cu_3_SbS_4_ and Cu_2_S (PDF Card n. 01-072-1071) as secondary phases, as also reported by Chetty et al. and Harish at al. [16,47]. The cross-section of the water-based resin coated THD after curing at 250 °C for 45 min (Figure 8) shows crystals of different shape and composition well dispersed in the silicone resin matrix, and no pores, cracks or other defects are visible at the coating/THD interface. 

**Figure 9:**
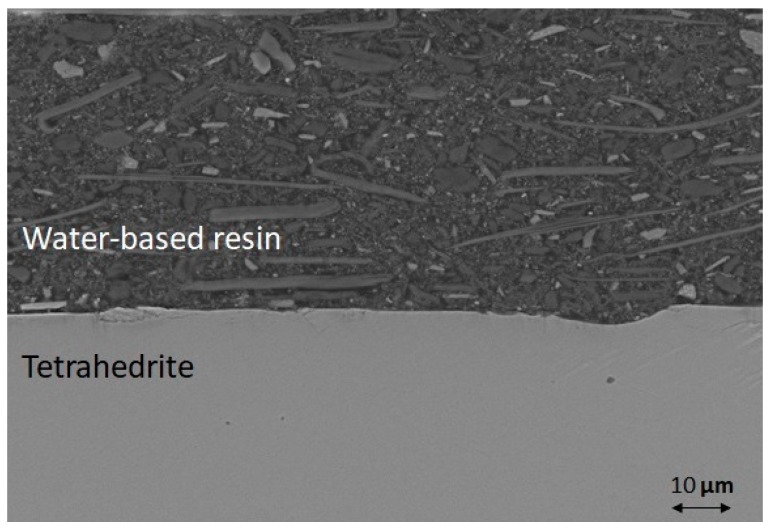
SEM image of cross-section of water-based resin coated THD after aging at 350 °C, dwelling time 48 h, in air.

The cross-sectional image of the water-based coated THD after ageing at 350 °C for 48 h (Figure 9) evidenced the absence of cracks within the coating, which is still well-adhered to the substrate. As can be seen in the SEM image, no evidence for the formation of oxidation layers was found at the coating/THD interface. Furthermore, XRD analysis (Figure 6d) shows that after the ageing at 350 °C for 48 h in air there were no apparent changes in the THD compared to the as-sintered sample, confirming that the hybrid coating provided an effective protection, inhibiting the oxidation of THD under thermal ageing.

**Figure 10:**
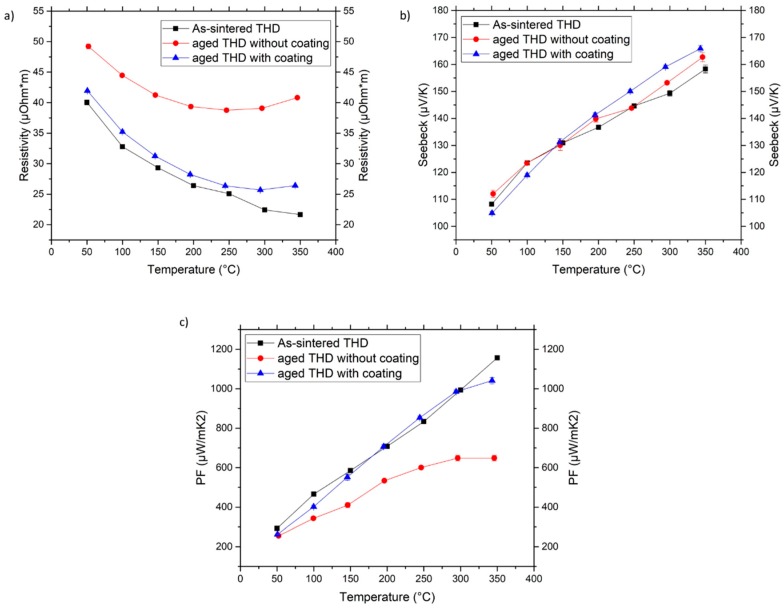
Temperature dependence of the (**a**) Electrical resistivity (**b**) Seebeck coefficient and (**c**) Power Factor of as-sintered THD and aged THD, with and without coating after ageing at 350 °C for 48 h.

A comparison between the properties of the as-sintered sample and the uncoated and coated counterparts after ageing at 350 °C for 48 h in air (Figure 10), further confirm the effectiveness of the coating. The Seebeck coefficients of the three samples did not show any differences, but the values of the aged THD with coating were slightly higher than those without coating, at least starting from 150 °C. The coating is able to prevent the increase in electrical resistivity noticed in the uncoated sample, as it maintains the original chemical composition. Consequently, the power factor of the uncoated sample suffers a significant reduction, while the coated sample maintains a similar value. 

## 4. Conclusions

The effectiveness of two hybrid protective coatings for Mg_2.1_Si_0.487_Sn_0.5_Sb_0.13_ and Cu_11.5_Zn_0.5_Sb_4_S_13_ thermoelectric materials was reviewed and discussed. 

The solvent-based resin significantly reduced the oxidation rate of magnesium silicide at 500 °C in air. Even with some imperfections (incomplete adhesion, few cracks within the resin after curing), the coated sample was not significantly oxidised after 120 h and the electrical properties were not severely modified. 

The water-based hybrid coating was effective at providing a barrier coating to avoid the oxidation. Consequently, the values of the power factor did not decrease in the presence of the hybrid coating, indicating that it is a promising candidate for protecting THD against high temperature oxidation in air.
